# Using epitope predictions to evaluate efficacy and population coverage of the Mtb72f vaccine for tuberculosis

**DOI:** 10.1186/1471-2172-11-18

**Published:** 2010-03-30

**Authors:** Lucy A McNamara, Yongqun He, Zhenhua Yang

**Affiliations:** 1 Department of Epidemiology University of Michigan, Ann Arbor, MI 48109, USA; 2 Department of Microbiology and Immunology University of Michigan, Ann Arbor, MI 48109, USA; 3 Unit for Laboratory Animal Medicine University of Michigan, Ann Arbor, MI 48109, USA; 4 Center for Computational Medicine and Bioinformatics, University of Michigan, Ann Arbor, MI 48109, USA

## Abstract

**Background:**

The Mtb72f subunit vaccine for tuberculosis, currently in clinical trials, is hoped to provide improved protection compared to the current BCG vaccine. It is not clear, however, whether Mtb72f would be equally protective in the different human populations suffering from a high burden of tuberculosis. Previous work by Hebert and colleagues demonstrated that the PPE18 protein of Mtb72f had significant variability in a sample of clinical *M. tuberculosis *isolates. However, whether this variation might impact the efficacy of Mtb72f in the context of the microbial and host immune system interactions remained to be determined. The present study assesses Mtb72f's predicted efficacy in people with different DRB1 genotypes to predict whether the vaccine will protect against diverse clinical strains of *M. tuberculosis *in a diverse host population.

**Results:**

We evaluated the binding of epitopes in the vaccine to different alleles of the human DRB1 Class II MHC protein using freely available epitope prediction programs and compared protein sequences from clinical isolates to the sequences included in the Mtb72f vaccine. This analysis predicted that the Mtb72f vaccine would be less effective for several DRB1 genotypes, due either to limited vaccine epitope binding to the DRB1 proteins or to binding primarily by unconserved PPE18 epitopes. Furthermore, we found that these less-protective DRB1 alleles are found at a very high frequency in several populations with a high burden of tuberculosis.

**Conclusion:**

Although the Mtb72f vaccine candidate has shown promise in animal and clinical trials thus far, it may not be optimally effective in some genotypic backgrounds. Due to variation in both *M. tuberculosis *protein sequences and epitope-binding capabilities of different HLA alleles, certain human populations with a high burden of tuberculosis may not be optimally protected by the Mtb72f vaccine. The efficacy of the Mtb72f vaccine should be further examined in these particular populations to determine whether additional protective measures might be necessary for these regions.

## Background

Although the Bacille Calmette Guérin (BCG) vaccine for tuberculosis (TB) is the most widely used vaccine worldwide, TB continues to be a tremendous public health problem [1]. A third of the world's population is estimated to be infected with *Mycobacterium tuberculosis *and 2-3 million people die of the disease each year [1,2]. Key among the reasons for the unabated spread of TB is the inability of the BCG vaccine to provide adequate protection against pulmonary TB in adults, the most contagious form of TB [1]. Developing an improved vaccine for TB, whether a replacement for BCG, a booster to the existing vaccine, or a vaccine specifically directed against latent TB, is of crucial importance in the battle to defeat the disease [3,4].

While several TB vaccine candidates have demonstrated protective efficacy in animal models and have proceeded to clinical trials in humans [1,3], even a successful clinical trial cannot guarantee that a vaccine can protect all members of the diverse worldwide human population against all variants of *M. tuberculosis*. One promising vaccine candidate is the Mtb72f subunit vaccine, a polyprotein composed of the *M. tuberculosis *proteins PepA and PPE18 [5]. The PPE18 antigen has been demonstrated to contain at least 10 epitopes [6] and the vaccine has been shown to provide protection against TB in cynomolgus monkeys [7]; it is currently under clinical trials in humans. The peptide sequence of Mtb72f, like that of many vaccines, is based on a laboratory strain of the pathogen whose antigens may differ from those found in variable clinical strains in immunologically critical ways [8]. Furthermore, the diversity of the Human Leukocyte Antigen (HLA) and other immune genes results in variable vaccine efficacy in different individuals even in the absence of pathogen variation [9]. While clinical trials include individuals of many HLA genotypes, genotype frequencies vary dramatically in different regions; genotypes that are common in TB-endemic regions may be underrepresented in clinical trial populations. It is therefore necessary to incorporate information on host and pathogen genetic diversity in vaccine design, development, and testing.

The PPE protein family of *M. tuberculosis *is a large, 69-member protein family with a currently unknown function [10]. Previous work by Hebert and colleagues [8] demonstrated that the PPE18 protein of Mtb72f, but not the PepA protein, had significant variability in a sample of clinical *M. tuberculosis *isolates. Such variation, however, would be important only if the variation were in regions of the protein that were vital for the human immune response to *M. tuberculosis*. To determine whether variation in these proteins might impact the efficacy of Mtb72f, we must consider interactions between the *M. tuberculosis *proteins and the human immune system.

Of the many genes involved in the immune response, the Major Histocompatibility Complex (MHC) genes - HLA in humans - are among the most crucial and yet most variable. MHC proteins present foreign peptide epitopes from intracellular (MHC Class I) and extracellular (MHC Class II) pathogens to CD8+ cytotoxic (Class I) or CD4+ helper (Class II) T cells to initiate the immune response. The Mtb72f vaccine was found to stimulate both CD4+ and CD8+ T cell-mediated responses in a mouse model [5], and the CD4+ T cell response in particular is thought to be essential for preventing *M. tuberculosis *infection [1,2]. There are thousands of HLA alleles [11], however, and variation in these alleles can significantly impact individual responses to vaccination [9].

Recently, several algorithms to predict the affinity of peptide sequences for various Class I and Class II MHC alleles have been developed [12,13] and several have been extensively validated [11-15]. A consensus approach incorporating three or more programs has been shown to increase the accuracy of MHC Class II epitope predictions [15,16]. To investigate the impact of host and pathogen variation on TB vaccine efficacy, we have used previously reported protein sequences from clinical isolates of *M. tuberculosis *[8] and *in silico *HLA epitope prediction programs to assess the protection offered by the Mtb72f subunit vaccine against diverse strains of *M. tuberculosis *in human populations suffering from a high burden of the disease. This investigation revealed that due to variation in both *M. tuberculosis *protein sequences and epitope-binding capabilities of different HLA alleles, certain human populations with a high burden of tuberculosis may not be optimally protected by the Mtb72f vaccine.

## Results

### Comparison of epitopes between the vaccine and clinical strains

To determine the impact of clinical strain variation on the protection offered by the Mtb72f vaccine, we examined the conservation of each nonamer sequence predicted to be a binding epitope compared to the conservation of every nonamer sequence in the vaccine. Eight epitope prediction programs were used to determine epitopes predicted to bind (Table 1). There was substantial variation in the number of nonamer sequences predicted to act as epitopes for each DRB1 allele by each prediction program (Table 2). However, when a list of all PPE18 nonamer cores predicted to bind at least one DRB1 allele was compiled using the epitope predictions for the five prediction programs that covered the greatest number of DRB1 alleles, 65% of these nonamers were unconserved. This percentage was significantly greater than the 60% of total PPE18 nonamer sequences that were unconserved (Binomial test, p < 0.0001). We also considered individual prediction programs' epitope binding predictions for single DRB1 alleles. For 41 out of 56 individual DRB1 allele/prediction program combinations, more than 60% of the epitopes predicted to bind were unconserved; for 14 of these, 80-100% were unconserved (Table 2). No nonamer sequences in the PepA protein were classified as unconserved epitopes.

**Table 1:** Attributes of and citations for the epitope prediction programs used

*Program*	*Prediction method*	*Unique features*	*Source paper (number of citations^1^)*	*URL*
ARB	Average relative binding matrices		[42] (59)	[43]
MHCPred	Partial least squares		[33] (46)	[44]
			[34] (30)	
			[35] (30)	
ProPred	Matrix-based	TEPITOPE matrices; requires key anchor residues	[45] (186)	[41]
			TEPITOPE: [30] (310)	
RankPep	Position Specific		[23] (119)	[47]
	Scoring Matrices		[46] (102)	
NetMHCII	Position Specific Scoring Matrices	Predicts epitopes of multiple lengths; uses SMM-align matrices	[37] (29)	[48]
SVRMHC	Support vector machine regression		[32] (16)	[49]
			[31] (23)	
Vaxign	Position Specific Scoring Matrices		[40] (0)	[50]
NetMHCIIpan	Artificial Neural Networks	Predicts multiple-length epitope binding to every sequenced Class II allele	[11] (3)	[51]

^1 ^Citation numbers are from Google Scholar

**Table 2:** Total number of PPE18 epitopes predicted to bind each allele by the five programs that predicted epitope binding for the largest number of DRB1 alleles and the number and percentage of unconserved epitopes

	No of total predicted epitopes					No of unconserved epitopes (*%*)				
*DRB1 Allele*	*ARB*	*ProPred*	*RankPep*	*NetMHCII*	*NetMHCIIpan*	*ARB*	*ProPred*	*RankPep*	*NetMHCII*	*NetMHCIIpan*
0101	133	17	30	104	77	83 (*62*)	9 (*53*)	20 (*67*)	61 (*59*)	46 (*60*)
0301	17	8	0	0	1	13 (*76*)	4 (*50*)	0 (*N/A*)	0 (*N/A*)	1 (*100*)
0401	33	12	33	18	7	20 (*61*)	8 (*67*)	23 (*70*)	13 (*72*)	7 (*100*)
0404	47	15	5	14	10	30 (*64*)	10 (*67*)	5 (*100*)	14 (*71*)	8 (*80*)
0405	29	13	2	14	13	20 (*69*)	8 (*62*)	1 (*50*)	9 (*64*)	9 (*69*)
0701	31	7	6	16	28	22 (*71*)	5 (*71*)	4 (*67*)	11 (*69*)	19 (*68*)
0802	8	7		0	1	7 (*87*)	6 (*86*)		0 (*N/A*)	1 (*100*)
0901	35		3	22	21	18 (*51*)		3 (*100*)	13 (*59*)	14 (*67*)
1101	16	13	15	5	4	12 (*75*)	10 (*77*)	6 (*40*)	3 (*60*)	3 (*75*)
1301		7	0		1		6 (*86*)	0 (*N/A*)		1 (*100*)
1302	18	5	0	24	2	13 (*72*)	4 (*80*)	0 (*N/A*)	16 (*67*)	2 (*100*)
1501	14	10	1	12	7	10 (*71*)	8 (*80*)	1 (*100*)	6 (*50*)	5 (*71*)

### Prediction of promiscuous epitopes

As promiscuous epitopes are frequently sought for epitope-based vaccines, we examined such epitopes, defining them as epitopes predicted to bind at least four of the twelve DRB1 alleles studied and classifying them as conserved or unconserved (Table 3). In all, fifteen promiscuous epitopes were predicted, ten from the PPE18 protein and five from PepA. Eight of these epitopes, including all of the PepA epitopes, were classified as conserved and the other seven as unconserved. Of note, the three PPE18 epitopes predicted to bind the largest numbers of epitopes (9, 6, and 6, respectively) were all classified as unconserved.

**Table 3:** Conservation of promiscuous epitopes

*Epitope core*	*Protein*	*DRB1 alleles predicted to bind*	*Conservation*
VRAMSSLGS	PPE18	0101, 0401, 0404, 0405, 0802, 0901, 1101, 1301, 1501	Unconserved
MILIATNLL	PPE18	0101, 0401, 0404, 1301, 1302, 1501	Unconserved
MVSMANNHM	PPE18	0101, 0401, 0404, 0405, 1301, 1302	Unconserved
YVAWMSVTA	PPE18	0101, 0401, 0404, 0405, 1101	Conserved
LLGQNTPAI	PPE18	0101, 0401, 0404, 1301	Conserved
ILIATNLLG	PPE18	0101, 0401, 0404, 1301, 1501	Unconserved
ISNMVSMAN	PPE18	0101, 0401, 0404, 0405, 1101	Unconserved
LFSAASAFQ	PPE18	0101, 0401, 0405, 0901	Conserved
YVMPHSPAA	PPE18	0101, 0401, 0405, 0802	Unconserved
VSMTNTLSS	PPE18	0101, 0401, 0404, 0405	Unconserved
VVGMNTAAS	PepA	0101, 0301, 0401, 0404, 0405, 0802, 1101, 1301, 1501	Conserved
VVLTNNHVI	PepA	0101, 0405, 0701, 0802, 1101, 1301, 1302, 1501	Conserved
VVGSAPAAS	PepA	0101, 0404, 0802, 0901, 1101, 1301	Conserved
FLGLGVVDN	PepA	0101, 0401, 0405, 1101	Conserved
LRGAGGLPS	PepA	0101, 0802, 1101, 1301	Conserved

### Prediction of DRB1 allele binding

**Figure 1:**
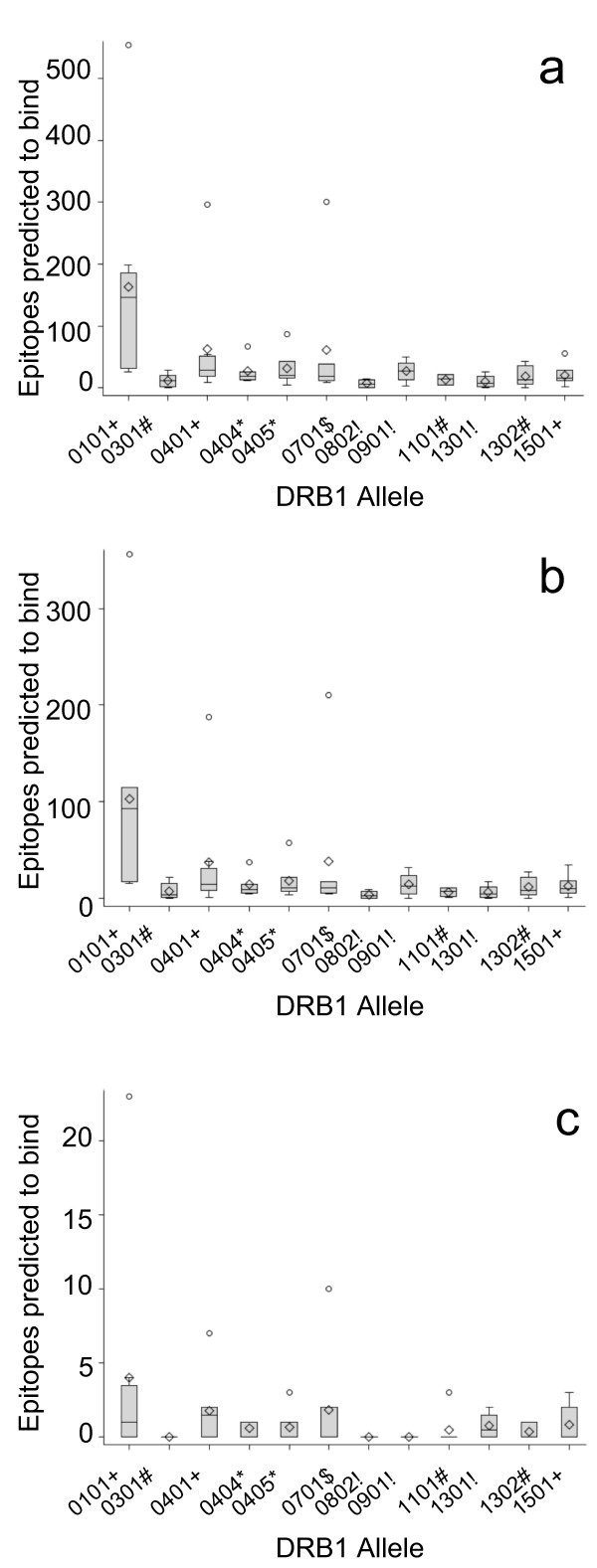
**Boxplots of CD4+ T cell epitope nonamer cores (y-axis) in the Mtb72f vaccine predicted to bind to each of the twelve selected DRB1 alleles (x-axis) by the eight chosen epitope prediction programs**. Epitopes found in the unmodified PepA protein but not in the vaccine have been excluded, as have epitopes found in the vaccine but not in the unmodified proteins. Boxes show the lower quartile, median, and upper quartile; whiskers show the minimum and maximum values excluding outliers (circles) and diamonds show the mean. The symbols next to the names of DRB1 alleles indicate the numbers of programs used to predict epitope binding to each allele: ! = 4, * = 5, # = 6, $ = 7, and + = 8. The highest outliers for alleles 0101, 0401, and 0701 on each chart are from MHCPred. **a**. Total vaccine epitope binding predictions. **b**. Vaccine epitope binding predictions for conserved epitopes only. Conserved epitopes are defined as epitope nonamer cores plus N- and C-terminal pentamer flanking sequences that are absent or mutated in no more than two of the variant strains sequenced. **c**. Epitopes in the vaccine but not in the native PPE18 or PepA proteins that are predicted to bind each DRB1 allele.

To find DRB1 alleles that might be especially well or poorly covered by the Mtb72f vaccine, we summed the PPE18 and PepA epitope predictions to find the number of total (Figure 1a) and conserved (Figure 1b) vaccine epitopes predicted to bind each DRB1 allele. DRB1*0101 was consistently predicted to bind an extremely large number of epitopes; a median of 147 total epitopes (range 26-554) and 93 conserved epitopes (range 16-356) were predicted to bind. By contrast, DRB1 alleles 0301, 0802, and 1301 were each predicted to bind a very small number of total or conserved vaccine epitopes. DRB1*0301 was predicted to bind a median of 11 total vaccine epitopes (range = 0-29) and four conserved vaccine epitopes (range = 0-22) while DRB1*0802 was predicted to bind a median of seven total vaccine epitopes (range = 0-15) and three conserved vaccine epitopes (range = 0-9). Finally, DRB1*1301 was predicted to bind a median of eight total vaccine epitopes (range = 0-26) and five conserved vaccine epitopes (range = 0-17). Similar trends were observed when total PPE18 epitopes, conserved PPE18 epitopes, and PepA epitopes predicted to bind each allele were evaluated separately (data not shown).

### Non-TB antigenic regions

Because the Mtb72f vaccine is a polyprotein composed of a single continuous amino acid chain incorporating the full PPE18 protein, the PepA protein separated into two pieces, an N-terminal His tag, and short amino acid insertions between the protein segments, several potential MHC Class II epitopes are found in the vaccine but not in the native *M. tuberculosis *proteins [5]. If these epitopes bound to DRB1 proteins, they could misdirect T cells to respond to non-TB epitopes rather than to epitopes found in the pathogen [17]. Non-pathogen epitopes make up a relatively high proportion of the epitopes in the Mtb72f vaccine compared to other vaccines, such as killed or live attenuated vaccines that do not contain artificial epitopes, and thus these epitopes might misdirect the immune response to Mtb72f in a manner not seen with other vaccines. We therefore evaluated the DRB1 binding of each potential MHC Class II epitope core found in the vaccine but not in the individual proteins. The maximum median number of non-TB vaccine epitopes predicted to bind to any of the DRB1 alleles was two (Figure 1c). Of the three DRB1 epitopes predicted to bind the smallest number of total or conserved vaccine epitopes (DRB1*0301, 0802, and 1301; Figure 1a and 1b), two were predicted to bind none of the non-TB epitopes and the third, DRB1*1301, was predicted to bind a median of one non-TB epitope (Figure 1c).

### Alleles of concern in regions with a high burden of TB

HLA DRB1 alleles of concern were defined as DRB1 alleles predicted to bind four or fewer conserved epitopes in the Mtb72f vaccine.  Based on these criteria, we found that the alleles of concern were DRB1*0301, 0302, 0403, 0411, 0802, 0803, 0807, 1202, 1401, 1403, 1404, 1405, and 1504 (Table 4). Of these alleles, only four (DRB1*0803, 0807, 1401, and 1404) were predicted to bind as many or more conserved as unconserved epitopes, while the remaining nine were predicted to bind fewer conserved than unconserved epitopes or (DRB1*0302) to bind no epitopes at all. Five additional alleles (DRB1*1101, 0901, 1402, 1502, and 1602) that were not predicted to bind particularly few conserved epitopes nevertheless bound at least as many unconserved as conserved epitopes (Table 4). We further examined whether any of these alleles were predicted to bind the non-TB epitopes in the vaccine. Although most of the alleles were not predicted to bind any of these epitopes, DRB1*0302, 0403, and 0411 were each predicted to bind to one non-TB epitope (data not shown).

**Table 4:** Epitope binding predictions for DRB1 alleles that are common in TB high-burden countries

*DRB1 Allele*^1^	*Median total (conserved) epitopes*	*N*^2^	*Association with tuberculosis (reference)*	*Tuberculosis-endemic populations where allele is highly prevalent*^4^
0101	122 (76)	9	NK^3^	Russian Chuvash, Aleuts and Tuva, Indian Islamic populations
0102	46 (30)	3	NK	Ethiopia (Amhara)
*0301*	11 (4)	6	Protective [29]	Brazil, northern China, Ethiopia (Oromo), northern and eastern India, northwestern Russia, Thailand (Bangkok), South Africa (Venda)
*0302*	0 (0)	1	NK	South Africa (Venda)
0401	29 (15)	8	NK	Chinese Inner Mongolian Evenki, Russian Chukchi, Eskimo, Koryak, Buryat, and Negidal populations
*0403*	11 (4)	1	NK	Russian Buryat and Nganasan populations
0404	16 (8)	6	NK	Brazil Kaingang indigenous population
0405	20 (11)	6	NK	Philippines
*0411*	7 (3)	1	NK	Brazil indigenous populations
0701	16 (9)	8	Protective [29]	Brazil, Northern and Eastern China, Democratic Republic of the Congo, Ethiopia, Indonesia (Java), Russian Bearian Island Aleuts, Russia Chuvash, Russia (Siberia), Thailand (Bangkok), Vietnam (Hanoi)
0801	9 (7)	2	Susceptible [29]	Russia (Kets)
*0802*	7 (3)	4	Susceptible [29]	Brazil indigenous populations, Russian Eskimos
*0803*	2 (1)	1	Susceptible [52]	China (Yunnan Province)
0804	7 (5)	2	NK	Brazil East Amazon indigenous populations
*0807*	2 (2)	1	NK	Brazil Guarani Kaiowa and Ticuna indigenous populations
0901	23 (10)	5	NK	Brazil Southeast Caucasian population, China, Russia (Siberia), Thailand, Vietnam (Hanoi)
1001	138 (77)	1	NK	Northern and eastern India, Russian Buryat population
1101	13 (5)	7	Susceptible [53]; Protective [29]	Northeast Brazil, Northern and central China, Democratic Republic of the Congo, northern India, Indonesia (Molucca and Nusa Tenggara), Russian Evenks, Nganasan, and Tuva populations, Zimbabwe (Harare)
1201	13 (7)	2	NK	China (Southern and Harbin), northeast India, Russia (Siberia)
*1202*	8 (2)	1	NK	Southern and central China, Hong Kong and Singapore, Indonesia, Philippines, Thailand, Vietnam (Hanoi)
1301	8 (5)	4	Protective [29]	Brazil, China (Xinjiang), Democratic Republic of the Congo, India (Andhra Pradesh), Russia (Kets, Khanty-Mansi), South Africa (Venda), Zimbabwe (Harare)
1302	14 (8)	6	Susceptible [53]; Protective [29,53]	Brazil Southeast Mulattos, Democratic Republic of the Congo, Ethiopia
1303	9 (5)	1	NK	Democratic Republic of the Congo, Northeast India
*1401*	5 (3)	1	Susceptible [29]	Southern China, Northern India, Philippines, Russian Nivkhi and Evenki, Udege, and Ulchi, Vietnamese Muong
1402	19 (8)	1	NK	Brazil Xavantes, Guarani, and Terena; Russian Chukchi, Eskimos, Koryaks, Nivkhi, and Udege
*1403*	2 (0)	1	NK	Chinese Drung, Russian Evenki and Kets
*1404*	3 (2)	1	NK	China Naxi and Lisu, India (Delhi)
*1405*	10 (3)	1	NK	Chinese Wa Population
1413	38 (19)	1	NK	Brazil Guarani M bya population
1501	15 (9)	8	Susceptible [29,54,55]	China, Hong Kong and Singapore, India, Indonesia, Russia (Siberia), Northeast Thailand
1502	12 (5)	2	Susceptible [29]	Chinese Jino population, India, Indonesia, Philippines, Thailand, Vietnam
1503	15 (8)	1	NK	Zimbabwe (Harare)
*1504*	6 (2)	1	NK	Chinese Nu and Va populations
1602	17 (6)	1	NK	Brazil Xavantes, Guarani, Kaingang, Terena, and Ticuna populations, Chinese Maonan and Miao populations, Northern Thailand, Vietnamse Muong

^1^Alleles of particular concern (median of four or fewer conserved vaccine epitopes) are highlighted with italics.

^2^N = number of programs able to predict epitope binding to that allele

^3^NK = None known

^4^Of the study populations included in the Allele*Frequencies in Worldwide Populations database [25]

### Populations of concern for reduced vaccine efficacy

Based on the alleles of concern determined through epitope binding predictions, we characterized each population in the Allele*Frequencies Database from a TB-endemic country as being of great, moderate, or lesser concern for reduced Mtb72f vaccine efficacy. Populations of moderate or great concern were those in which alleles that bound relatively few conserved vaccine epitopes were particularly common (see definitions above). The populations of moderate concern are the Ticuna population of Brazil; China's Shanxi Province and Maonan, Kazak, Bai, Lahu, Naxi, Jino, and Yai ethnic minorities throughout the country; a population in Delhi, India; the Philippines; the Venda population of South Africa; a population in Bangkok, Thailand; and Vietnam. The populations of great concern are the Kaingang and East Amazon indigenous populations of Brazil; the Xinjiang Uyghur Autonomous Region of China as well as Yunnan Province's Drung, Va, Lisu, Naxi, and Nu ethnic minorities; and Indonesian Java.

## Discussion

Despite the striking variation in the protective efficacy of the BCG vaccine for TB observed among different world populations[18], the joint impact of host and pathogen variation on novel TB vaccine candidates has never been characterized. Using epitope prediction programs, we investigated the impact of clinical variations in the Mtb72f TB vaccine components, the PPE18 and PepA proteins, on epitope binding to alleles of the Class II HLA DRB1 gene. We identified conserved and unconserved promiscuous epitopes in the PPE18 and PepA proteins using the clinical variants described previously [8] and determined that while 60% of potential CD4+ T-cell epitopes in the PPE18 protein were unconserved, 65% of the actually predicted T-cell epitopes in this protein were unconserved. We furthermore found several DRB1 alleles that bound few vaccine epitopes overall and others that bound predominantly unconserved or non-TB epitopes, allowing us to determine individual genotypes as well as broader populations where the Mtb72f vaccine may not offer maximum protection.

Our finding that 60% of potential CD4+ T-cell epitopes in PPE18 are unconserved is consistent with previous research that has found the PPE gene family to be particularly variable among tuberculosis isolates [19]. The significant increase in the proportion of unconserved T-cell epitopes among the actually predicted epitopes compared to the potential epitopes (all nonamer sequences in the vaccine) likely reflects the selective pressure that the immune system places upon the bacterium to alter antigenic protein regions. Based on the clinical protein sequences previously described [8], no potential epitopes in the PepA protein were classified as unconserved. Although to our knowledge no other studies have compared PepA protein conservation to that of other *M. tuberculosis *proteins, previous findings that PepA is highly conserved among *Mycobacterium *species [20] suggest that PepA may be a relatively well-conserved protein. The high conservation of the PepA protein even under selective pressure from the immune response may indicate that little variation in this protein is compatible with protein function, and thus PepA may be a particularly good vaccine target [21].

We also examined the conservation of promiscuous epitopes. Epitope promiscuity is commonly evaluated in the generation of epitope-based vaccines [22-24], but since they are antigenic, promiscuously binding epitopes should be selected against by a large range of host immune systems. We would therefore expect to find many unconserved promiscuous epitopes. This was found to be the case: seven of the ten promiscuous epitopes predicted for PPE18 were unconserved. However, all five of the promiscuous PepA epitopes were conserved, and thus these epitopes could be particularly good candidates for epitope-based vaccines.

Although the majority of the promiscuous PPE18 epitopes identified were found to be unconserved, it is possible that these epitopes might nevertheless provide protection against the bacterium if they are found in other *M. tuberculosis *proteins. We therefore investigated whether these epitopes were found in two proteins (PPE19 and PPE60) that are closely related to PPE18. This analysis revealed that only three of the promiscuous epitopes were present in all three proteins. The remaining seven were found only in PPE18.

Our finding that three of the twelve DRB1 alleles selected, DRB1*0301, DRB1*0802, and DRB1*1301, were predicted to bind few total and conserved epitopes in the Mtb72f vaccine suggests that the vaccine might not be maximally protective in people who have one or more of these alleles. By contrast, DRB1*0101 was consistently predicted to bind far more total (median 147) and conserved (median 93) vaccine epitopes than any other DRB1 allele (median 16 total or 9 conserved), suggesting that individuals with this allele might be particularly well protected by the vaccine. DRB1*0101 is a common allele in the US and Belgium, where Phase I clinical trials for the Mtb72f vaccine were conducted, but unfortunately is rarer in Brazil, China, Indonesia, and many other countries where TB is endemic according to the Allele*Frequencies in Worldwide Populations database [25]. Therefore, while the vaccine may be effective in the US and Belgium, it may not be equally effective in other regions.

Our analysis of all the DRB1 alleles in the Allele*Frequencies in WorldWide Populations database [25] that were found to be one of the top three most common alleles in any population in any of the twenty-two countries designated as TB high-burden countries by the WHO [26] generated a list of the alleles of concern in high-burden TB countries. These alleles include DRB1*0301, 0302, 0403, 0411, 0802, 0803, 0807, 1202, 1401, 1403, 1404, 1405, and 1504. Although the epitope predictions for these alleles should be treated with more caution, as predictions for most could be generated by only one or two prediction programs, the importance of these alleles merits their consideration.

While the number of epitopes capable of binding to a particular MHC allele does not necessarily indicate how strong the immune response will be in that MHC background [27], the fact that many of these alleles bind very few conserved epitopes is of concern for two reasons. First, the analysis conducted here includes only predictions of epitope binding to the MHC molecule and not the cellular processing that must take place before MHC presentation occurs. Because of this processing, many potential antigenic peptides will not be generated *in vivo*. For MHC alleles to which few epitopes are predicted to bind, it is possible that none of the potentially binding epitopes will be generated *in vivo *and thus that no epitopes in the vaccine will be presented on DRB1 proteins. Furthermore, for some DRB1 alleles the number of conserved epitopes predicted to bind is much smaller than the number of unconserved epitopes predicted to bind. In the immune response to a vaccine or pathogen, a single or small number of epitopes is usually immunodominant [27] even though many epitopes could potentially bind to the MHC alleles in question. For MHC alleles that are predicted to bind many fewer conserved than unconserved epitopes, it is likely that the immune response to the vaccine would be dominated by responses to unconserved epitopes and therefore would not be optimally protective against all *M. tuberculosis *strains. Most (8/13, ~62%) of the alleles of concern were predicted to bind at least as many unconserved as conserved epitopes, and one was predicted to bind no conserved or unconserved epitopes at all. Only five of the 21 alleles predicted to bind more than four conserved epitopes were predicted to bind at least as many unconserved as conserved epitopes. Individuals with these alleles - DRB1*1101, 0901, 1402, 1502, and 1602 - might also be less efficiently protected by the Mtb72f vaccine.

Because Mtb72f is a polyprotein containing several non-TB potential epitopes at the junction of the PPE18 and PepA proteins and at the N-terminus of the polyprotein, we investigated whether epitopes present in the Mtb72f vaccine but not in the native PPE18 and *PepA *proteins might misdirect the immune response upon immunization. Fortunately, most of the alleles of concern noted above did not bind any of the non-TB epitopes in the vaccine, but DRB1*0302, 0403, 0411, and 1301 were each predicted to bind to a median of one non-TB epitope. This finding is of particular concern for DRB1*0302, a common allele in South Africa, because it was not predicted to bind any of the protective epitopes (conserved or unconserved) in the vaccine. The DRB1-mediated immune response to Mtb72f in people with this allele might thus be misdirected against epitopes that are found in the vaccine but not in TB and thus this portion of the immune response would not be protective. As South Africa is one of the sites for the Phase II clinical trials of the Mtb72f vaccine, it would be beneficial to collect immunological data from vaccine recipients in order to determine whether the vaccine is effective in persons with the DRB1*0302 allele.

While this analysis should be useful for assessing population coverage of the Mtb72f vaccine, it is important to recognize the limitations inherent in the use of epitope prediction programs. No epitope prediction program is perfectly accurate, and there is substantial disagreement among the epitopes predicted by each program [14,15]. We increased the accuracy of our analysis by using multiple prediction programs for each DRB1 allele when possible, but there are nevertheless likely to be differences between the predicted and actual epitopes for many alleles. However, as we obtained good agreement among programs as far as which alleles were predicted to bind relatively many or few vaccine epitopes, the lists of alleles and populations of concern would not likely be substantially changed by improvements in the prediction programs or experimental confirmation.

Although we compared epitope predictions across eight different programs, these programs are not perfectly comparable. Several of the programs, such as Vaxign, provided information on peptide affinity predictions only for epitopes predicted to bind each allele. As we could not obtain affinity information for peptides predicted not to bind, we were unable to evaluate the binding threshold to which the prediction program was automatically set. Furthermore, the binding cutoffs used by several of the programs likely differ from the IC_50 _≤ 500 nM cutoff that we imposed on programs generating IC_50 _predictions. However, as the number of epitopes predicted by programs with non-IC_50_-based cutoffs generally though not always fell within the range of programs that did use the IC_50 _cutoff, it is unlikely that the differing thresholds among programs severely skewed the results.

Finally, it should be noted that the HLA allele frequencies in different populations [25] that were utilized to generate the list of alleles of concern were in some cases generated through only a few small studies. It is likely that further study of HLA genotypes would alter our predictions of which populations might be more or less effectively protected by the Mtb72f vaccine.

## Conclusions

We conclude that the Mtb72f vaccine may be less protective in certain populations that suffer from a high burden of TB and thus that additional protective measures may be needed in these populations in addition to this promising vaccine candidate. While the findings from this *in silico *analysis should be verified with immunological studies, this analysis complements *in vitro *and *in vivo *experimentation by vastly expanding the range of host and pathogen factors that can be examined and incorporating an analysis of many more genotypes than a laboratory or even clinical study could include. Furthermore, this type of analysis can be used to aid rational selection of populations in which to conduct clinical trials for vaccines. This method of vaccine evaluation could also be usefully extended to other subunit vaccine candidates for TB, as well as vaccine candidates for HIV and other diseases, and might provide a means of comparing different vaccines to select the best vaccine to use in a given population. However, continued improvements to epitope prediction software, refinement of programs to predict pre-presentation epitope processing, and further study of the HLA genotypes of world populations could help to increase the accuracy and utility of such vaccine evaluations.

## Methods

### Allele selection

Although both Class I and Class II-mediated T cell immunity are important in *M. tuberculosis *infection, we focused on alleles of the Class II MHC DRB1 gene for several reasons. First, Class II MHC molecules are the ones that interact with CD4+ T helper cells, which stimulate macrophages to kill phagocytosed pathogens. As *M*. *tuberculosis *inhabits macrophages, CD4+ T cell-mediated immunity is of particularly crucial importance for preventing and clearing *M. tuberculosis *infection (reviewed in [2]). DR alleles were examined because of the relative abundance of data on epitope binding to DR alleles; for TB more than 90% of known Class II *M. tuberculosis *epitopes bind to DR antigens [28]. Finally, the DRB1 gene was studied because DRB1 proteins are typically expressed at five-fold greater levels than are the DRB3, DRB4, or DRB5 genes [29]. To ensure high prediction accuracy, our initial analysis focused on the twelve DRB1 alleles for which epitope binding predictions were available from at least four of the eight epitope prediction programs used. These include DRB1*0101, 0301, 0401, 0404, 0405, 0701, 0802, 0901, 1101, 1301, 1302, and 1501. In addition, for each population in the Allele*Frequencies in Worldwide Populations database [25] that was from the twenty-two TB high burden countries identified by the WHO [26], we determined the three most common DRB1 alleles in that population and evaluated epitope binding predictions for each of these DRB1 alleles. These additional alleles were DRB1*0102, 0302, 0403, 0411, 0801, 0803, 0804, 0807, 1001, 1201, 1202, 1303, 1401, 1402, 1403, 1404, 1405, 1413, 1502, 1503, 1504, and 1602.

### Epitope prediction

Epitope prediction programs use several different types of prediction algorithms, including matrix-based methods (e.g. the TEPITOPE matrices [30]), artificial neural networks (e.g. NetMHCIIpan [11]), support vector machine regression methods (e.g. SVRMHC [31,32]), and partial least squares methods (e.g. MHCPred [33-35]). In addition to these different methods, some programs (e.g. MHC-BPS [36], NetMHCII [37]) can incorporate peptide length as well as peptide sequence into their prediction of peptide-MHC affinity. There is conflicting data as to which epitope prediction program provides the most accurate predictions for binding to each MHC molecule [13,14], but consensus approaches have been shown to increase prediction accuracy [15,16]. We therefore used eight online epitope prediction programs to evaluate the number of epitopes in the Mtb72f vaccine predicted to bind each human MHC Class II molecule of interest. To select programs for this study, a comprehensive list of the more than a dozen freely available epitope prediction programs for human Class II MHC alleles was generated through a search of the literature. We then selected programs that either explicitly predicted which peptides were predicted to bind each MHC molecule or predicted the half maximal inhibitory concentration (IC_50_) for each peptide, as binding predictions could be inferred directly from the IC_50 _values using the criterion that binding peptides usually have an IC_50 _≤ 500 [38]. Because we required discrimination of binding and non-binding peptides, several MHC Class II epitope prediction programs that neither explicitly predicted binding peptides nor provided an IC_50 _score for peptides, including SYFPEITHI, MHC2Pred, and PeptideCheck, were excluded.

The remaining programs were screened to ensure that each used a distinct epitope prediction algorithm. Furthermore, to maintain some consistency among alleles, we used only programs that could predict binding to at least three human Class II MHC alleles. MHC class II molecules typically bind peptides of length 10-30. However, a 9 amino acid binding core, or nonamer, is sufficient to bind to a MHC class II molecule [39]. We included only programs that either predicted only nonamer peptide binding or explicitly predicted the nonamer core when predicting the binding of longer peptides to ensure that the predictions from each of the programs used could be directly compared, since many of the epitope prediction programs could predict binding only for nonamer sequences (Table 1). Most of these programs have been frequently used for various types of epitope research; Vaxign is a recently developed program of epitope prediction specifically targeted for vaccine development [40].

Binding cutoffs were next determined for each program. For programs that explicitly predict binding epitopes (RankPep and Vaxign), the default cutoffs were used. For programs that assigned IC_50 _or -logIC_50 _values to each epitope (ARB, MHCPred, NetMHCII, NetMHCIIpan, and SVRMHC), binders were assigned as those epitopes with IC_50 _≤ 500 (recommended in [38]). Finally, for ProPred, the recommended criterion of classifying peptides with binding scores within the top 3% of all natural peptides as probable binders was used [41].

Each portion of the Mtb72f polyprotein sequence [5], PPE18 and the two sections of PepA, was entered separately into each prediction program and epitope predictions for the DRB1 alleles of interest were acquired. Binding predictions were also generated for all potential epitopes from the vaccine that were not in either of the two original proteins, including epitopes at the junctions of the two proteins in the polyprotein sequence and those from the N-terminal poly-His tag.

### Comparison of epitopes between vaccine and clinical strains

A previous study from our laboratory found that there was substantial variation in the PPE18 sequences and limited variations in the PepA sequences in a sample of clinical isolates from Turkey and Arkansas compared to those in the H37Rv laboratory reference strain [8]. In this study, we examined the effect of this clinical sequence variation on Mtb72f epitope binding. For each nonamer amino acid residue sequence in each protein, the number of unique clinical variants with a change in the nonamer sequence or in the N- or C-terminal pentamer flanking sequence was calculated. This epitope conservation data was then combined with our epitope predictions to determine whether epitopes predicted to be immunogenic in a certain DRB1 background would protect the host against a wide range of clinical *M. tuberculosis *strains.

### Data analysis

Epitope prediction and conservation data were imported into SAS version 9.2 (SAS Institute, Cary, NC) and compiled to determine conservation of each predicted epitope. Criteria for classifying epitopes as conserved or unconserved were determined by finding local minima in distributions of the proportions of clinical variant strains in which each epitope was conserved. This process resulted in a definition of unconserved epitopes as vaccine epitopes that were absent or mutated in three or more of the twenty-seven clinical variants of the PPE18 antigen found previously among a total of 225 clinical isolates obtained from Arkansas, United States and Malatya, Turkey [8].

The numbers of PepA, PPE18, and unconserved PPE18 epitopes predicted to bind each DRB1 allele were calculated to find alleles to which particularly few epitopes were predicted to bind. As all PepA nonamers were considered conserved, we did not analyze unconserved PepA epitopes separately. With the exception of NetMHCIIpan, each epitope prediction program could generate predictions for only a subset of the twelve alleles studied, and therefore a different subset of prediction programs was used to predict epitope binding to each allele. To ensure that any trends in the number of epitopes predicted to bind each allele was not due solely to the different group of prediction programs used to predict epitopes for each allele, several methods of adjusting the epitope predictions by the characteristics of the particular programs used were attempted but were not found to alter the observed trends (data not shown).

Further analyses were conducted to determine how many of the twelve commonly-predicted DRB1 alleles each epitope was predicted to bind. A consensus approach was used to define promiscuous epitopes, with an epitope considered "predicted to bind" to DRB1 alleles DRB1*0101, 0401, 0404, 0405, 0701, 0901, 1101 and 1302 if at least half of the epitope prediction programs available for the allele in question predicted epitope binding. For DRB1*0301, 0802, 1301, and 1501, there was reduced agreement among programs and fewer epitopes predicted overall; therefore epitopes were considered "predicted to bind" if at least 42% (1501), 33% (0301), or 25% (0802, 1301) of the epitope prediction programs available for that allele predicted that the epitope would bind.

The overall allele frequency of each DRB1 allele in TB-endemic populations was also considered to determine whether the Mtb72f vaccine would be equally effective in all populations considered. We characterized each population in a high burden TB country in the Allele*Frequencies Database as being of great, moderate, or lesser concern of reduced Mtb72f vaccine efficacy. Populations of moderate concern were defined as those where two of the three most common DRB1 alleles were predicted to bind a median of four or fewer conserved vaccine epitopes or the single most common DRB1 allele was present at a frequency of greater than 0.275 (a local minimum in the allele frequency distribution) and predicted to bind a median of four or fewer conserved vaccine epitopes. Populations for which all of the three most common DRB1 alleles were predicted to bind a median of four or fewer conserved vaccine epitopes or the single most common DRB1 allele was present at a frequency of greater than 0.495 (a local minimum in the allele frequency distribution) and predicted bind a median of four or fewer conserved vaccine epitopes were categorized as populations of great concern. All other populations were classified as populations of lesser concern.

## Authors' contributions

LM compiled epitope prediction and allele frequency data, conducted statistical analyses, interpreted the data, and drafted the manuscript. YH participated in the design of the study and reviewed the version of the manuscript to be submitted. ZY conceived of the study, participated in its design, and provided critical revision of the manuscript. All authors read and approved the final manuscript.
